# Polypoid Basal Cell Carcinoma on the Nose Tip

**DOI:** 10.1155/2022/4087202

**Published:** 2022-06-24

**Authors:** Mutsuki Hirakawa, Yuki Ishikura, Taketoshi Futatsuya, Reimon Yamaguchi, Akira Shimizu

**Affiliations:** Department of Dermatology, Kanazawa Medical University, Uchinada, Ishikawa, Japan

## Abstract

Basal cell carcinoma (BCC) is usually seen on the face as a pigmented nodule. We herein report a patient who presented with a polypoid BCC on the nose tip. Clinically, we suspected adnexal tumor; however, the findings of dermoscopy were consistent with that of BCC. Although the tumor was excised at the stalk, it was completely resected. Since the clinical manifestation was characteristic, we reviewed previously reported polypoid BCCs and found that this tumor can occur at any site, including the trunk and inguinal region, which are not preferential sites for ordinary BCC. There have been no reports of polypoid BCC on the nasal tip. The initial diagnoses varied, including adnexal tumors, and dermoscopic examinations proved useful for suspecting polypoid BCC. Histopathologically, the tumor cells in the resected specimens were within the polypoid area. Although BCC is a common tumor, polypoid BCC has distinct clinical features, and we should keep this rare subtype in mind.

## 1. Introduction

Basal cell carcinoma (BCC) is one of the most common cancers of the skin. It is seen on skin that is frequently exposed to sunlight. BCC has various clinical appearances and histological features. Some forms of common BCC, such as superficial, nodular, morphoeic, and ulcerated, are clinically recognized [1]. BCC rarely shows a pedunculated form, except for fibroepithelioma, which presents with a fibroma-like manifestation and is located on the back. In addition, polypoid BCC, which differs from fibroepithelioma, was reported by Megahed [2].

We herein report a patient with polypoid BCC presenting with a cystic and pedunculated nodule on the nose tip.

## 2. Case Report

**Figure 1:**
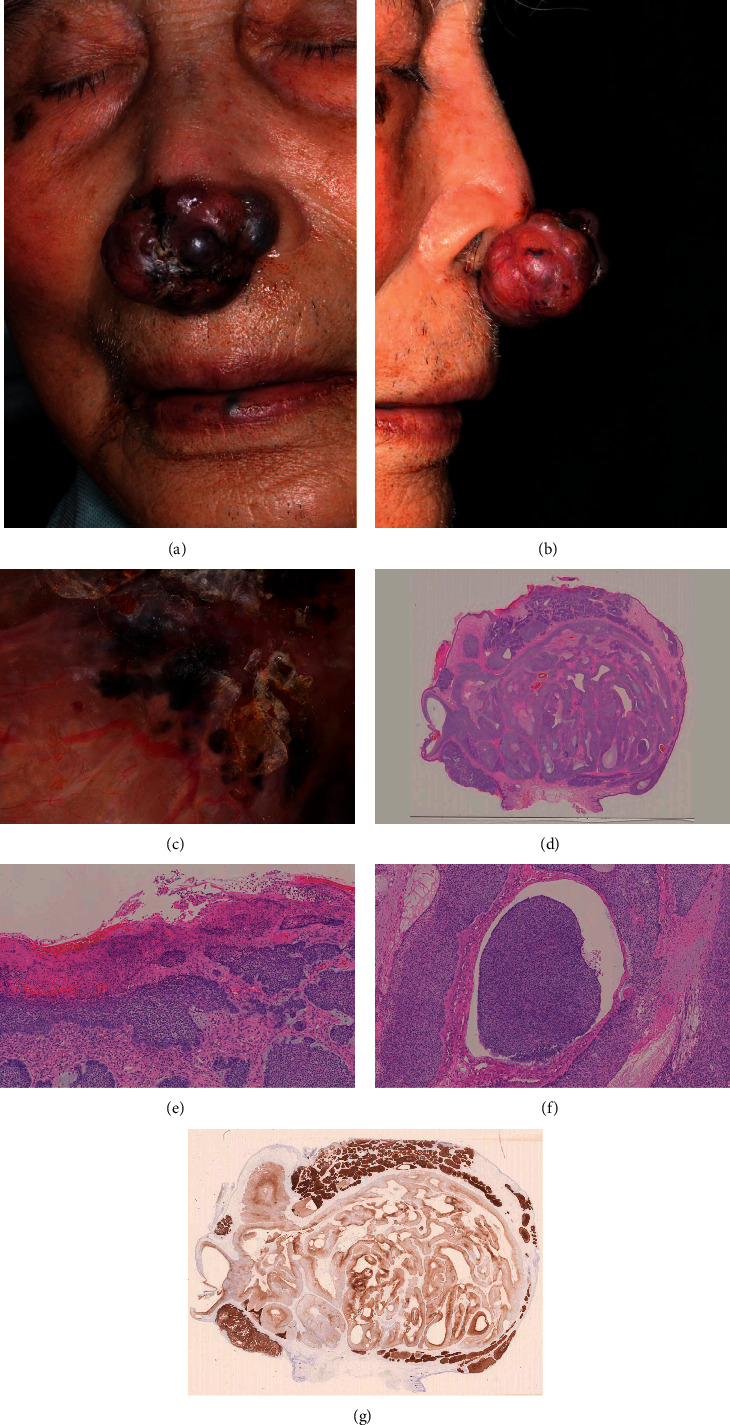
Clinical manifestation and histopathology. (a) A polypoid nodule was seen on the nose tip. (b) The lateral view shows a stalk on the nose. (c) Dermoscopy shows arborizing vessels and pigmented ovoid nests. (d) A low-power view showed basophilic nests in the dermis (hematoxylin and eosin staining, ×20). (e) A high-power view of the upper square showed basaloid cells with palisading (hematoxylin and eosin staining, ×200). (f) A high-power view of the lower square showed cystic spaces (hematoxylin and eosin staining, ×200). (g) Immunostaining with Ber-EP4 is more strongly positive in shallow lesions.

A 104-year-old male patient was referred to us with a tumor on the nose tip. The nodule had been noted for decades but had rapidly grown over the past two months. An elastic, hard, 40 × 25 × 15-mm, pedunculated red-black nodule with a smooth surface was found on the nose (Figures 1(a) and 1(b)). At first, we suspected an adnexal tumor rather than BCC based on its clinical appearance. However, a dermoscopy examination clearly showed arborizing vessels and black dots on its lower surface (Figure 1(c)), and a biopsy specimen revealed the nodular and cystic structure of basaloid cells, which was consistent with BCC.

The tumor was resected at the base of the stem, and the defect was cauterized with monopolar cautery. Histopathologically, there were two parts to this lesion, and a nodular and adenoid structure of basaloid cells was observed in the dermis (Figure 1(d)). The nuclei of the peripheral cell layer of the tumor masses were arranged in a palisade pattern. A cleft between the tumor and stroma was observed (Figure 1(e)). Cystic spaces containing mucinous and hyalinized tissues were also observed. There were some areas of adenoid differentiation and pseudocyst formation due to tumor necrosis (Figure 1(f)). Comparing the shallow lesions with the deep lesions, the deep lesions were marked by edema and myxomatous changes between tumor cells. Furthermore, immunohistochemical Ber-EP4 staining which is a useful marker for the diagnosis of neoplasms with follicular germinative differentiation was positive. The staining was more strongly positive in shallow lesions than in deep lesions (Figure 1(g)). ki67-positive cells were not clearly different between shallow and deep lesions, and EMA was negative (data not shown).

We finally diagnosed the patient with polypoid BCC on the nose tip. Two months later, the ulcer was epithelized by topical treatment. No recurrence has been seen for three months since excision.

## 3. Discussion

Megahed [2] proposed polypoid BCC as a new clinicopathological variant of nodular BCC in 1999 based on a few case reports. The author characterized BCC as follows: polypoid appearance, female predominance, and tumor restriction within the polypoid area. Our search identified 22 previously reported cases of polypoid BCC described in the English literature (Table 1) [2–18]. In our search, Gaughan [3] reported a giant pedunculated BCC on the forearm which arose from burn scar. Following that report, four more cases had been reported before Megahed's publication [2]; however, the characteristics of polypoid BCC were not fully described (Table 1). Among the 22 patients, there were 9 men and 13 women. The mean age was 58 years old (20–88, median 57 years old) which was younger than seen with ordinary BCC. The average size of the polyps was 28 mm. Despite the large size, most of the lesions displayed well-circumscribed nodules without any aggressive infiltrative pattern. The anatomic distribution of these polypoid BCC was distinctive: 5 (21%) on the scalp; 6 (25%) in the genital/perianal/buttock region; 3 (13%) on the trunk; 4 (17%) on the face; 2 (8%) in the periotic region; and 3 (13%) on the extremities. Typically, BCCs are commonly found on sun-exposed areas; however, polypoid BCC preferentially occurs on the scalp, genital areas, and back or buttock area. Polypoid BCC on the nose tip, as in our case, has never been reported. Adachi et al. [19] reviewed nine cases of Japanese polypoid BCC from their institution and noted that the polypoid BCC tumor was larger than other nodular BCCs, and the tumor site varied, including sites at the upper extremities, lower extremities, and genital area.

The manifestation is characteristic, and it is sometimes difficult to suspect BCC clinically. Seven cases of the reports stated that the initial clinical diagnosis was adnexal tumor or neurofibroma rather than BCC (Table 1). Our case clinically resembled adnexal tumor, but the findings of dermoscopy were consistent with BCC. Dermoscopy can be a valuable technique for distinguishing polypoid BCC from other pedunculated tumors. Some cases have demonstrated additional characteristics of polypoid BCC. Sahin et al. [4] described a case of keratotic BCC with polypoid manifestation. Wang et al. [5] reported multiple polypoid BCCs in a patient with basal cell nevus syndrome.

Histopathologically, 15 cases of polypoid BCC have shown the nodular type. Some cases also showed an adenoid/cystic structure histopathologically. The tumor nest was restricted to the polyp, which is important information for surgical excision. However, Kim et al. [13] described a case of polypoid BCC in the lower back, which progressed to pulmonary metastasis and death. Notably, in that case, the tumor was restricted to the polyp; however, it was the largest among the reported polyploid BCCs (Table 1).

The mechanism underlying the formation of polypoid BCC is unclear. Clothing-associated stimuli may be a cause, or there may be anatomical reasons, such as a rare fat condition. As Morita et al. [6] speculated, there are two patterns: preexisting lesions, such as fibroma and sebaceous nevus, and de novo lesions.

In our case, the positivity of the Ber-EP4 immunocytochemistry was stronger in shallow lesions than in deep lesions, suggesting that BCC is composed of two distinct tumour components. In our case, therefore, it was assumed that the shallow lesions developed first and that the rapid growth of the deeper mucinous lesions pushed the shallow lesions upwards. In addition, since the nose tip is located just above the bone, the rapid increase of the mucinous lesion may have resulted in this characteristic polypoid shape.

More cases of polypoid BCC need to be collected to clarify the characteristics.

## Figures and Tables

**Table 1:** Clinical and pathological summary of PBCC.

No	Year	Author	Age	Sex	Location	Size (mm)	Initial diagnosis	DS	Histopathology	TR	Others
1	1969	Gaughan^3^	68	F	forearm	50 × 40 × 25	ND	ND	ND	ND	Arising from burn scar
2	1985	Love^16^	64	M	Back	50 × 20	ND	ND	Adenoid	Yes	
3	1996	McElroy^17^	56	M	Shoulder	70 × 40	ND	ND	Nodular/Adenoid	ND	
4	1985	Morimoto^18^	69	M	Buttock	10 × 15	Pedunculated nevus, soft fibroma, neurofibroma	ND	Nodular/Adenoid	Yes	Deep margin positive
5	1995	Foley^19^	62	F	Eyelid	ND	ND	ND	ND	ND	Keratotic lesion
6	1999	Megahed^2^	87	F	ear	10 × 8	BCC, adnexal tumor, amelanotic melanoma	ND	Nodular	Yes	
7	1999	Megahed	76	F	Scalp	20 × 10	Adnexal tumor, verruca seborrheic,	ND	ND	Yes	
8	1999	Megahed	52	F	Scalp	15 × 10	Adnexal tumor, pedunculated dermal nevus	ND	ND	Yes	
9	1999	Megahed	20	F	Scalp	13 × 8	BCC, syringocystadenoma papilliferum, angioma developing on a nevus sebaceous	ND	Nodular	Yes	
10	2001	Morita^7^	33	F	Scalp	15 × 17	Pigmented nevus, fibroma	ND	Nodular/cystic	Yes	
11	2003	Sahin^5^	56	F	Inguinal region	40 × 30 × 25	Adnexal neoplasm	ND	Nodular	ND	Keratotic lesion
12	2004	Misago^8^	88	F	Buttock	35 × 20	PBCC, melanoma, adnexal neoplasm	ND	Nodular	Yes	
13	2006	Wang^6^	56	M	Perineum	21 × 15	PBCC, acrochordon, nevus	ND	Nodular	Yes	Multiple lesions, BCNS
14	2007	Sakai^9^	70	M	Eyelid	7	Neurofibroma, pigmented nevus	ND	Nodular	Yes	
15	2007	Choi^10^	56	F	Inguinal region	25 × 20	Pyogenic granuloma	ND	ND	Yes	
16	2007	Choi	69	F	calf	20 × 20	Melanoma, seborrheic keratosis	ND	Nodular	Yes	
17	2007	Choi	28	M	Scalp	15 × 10	Pyogenic granuloma	ND	ND	Yes	
18	2008	Feito-Rodriguez^11^	7	M	face, neck	2 (mean)	BCC	done	Nodular	Yes	Multiple lesions, BCNS
19	2008	Ouchi^12^	54	M	Scrotum	17	ND	ND	Nodular/cystic	Yes	
20	2008	Yadav^13^	57	F	Cheek	30 × 20	BCC, pyogenic granuloma,	ND	Nodular/Adenoid	ND	
21	2010	Kim^14^	61	M	Lower back	86 × 52 × 10	ND	ND	Nodular/Death	Yes	Metastasis
22	2017	Yildiz^15^	60	F	Postauricular region	7 × 5	BCC	done	Nodular	Yes	
23	2021	Present report	104	M	Nose tip	40 × 25 × 15	BCC, adnexal tumor	done	Nodular/Adenoid	Yes	

BCC, basal cell carcinoma; PBCC, polypoid BCC; DS, dermoscopy; TR, tumor restriction within the polypoid area; ND, not described; BCNS, basal cell nevus syndrome.
